# Invasive pulmonary mucormycosis: rare presentation with pulmonary eosinophilia

**DOI:** 10.1186/s12890-017-0419-1

**Published:** 2017-04-28

**Authors:** Taizou Hirano, Mitsuhiro Yamada, Kei Sato, Koji Murakami, Tokiwa Tamai, Yoshiya Mitsuhashi, Tsutomu Tamada, Hisatoshi Sugiura, Naomi Sato, Ryoko Saito, Junya Tominaga, Akira Watanabe, Masakazu Ichinose

**Affiliations:** 1 Department of Respiratory Medicine, Tohoku University Graduate School of Medicine, 1-1 Seiryo-machi, Aoba-ku, Sendai, 980-8574 Japan; 2 Department of Anatomic Pathology, Tohoku University Graduate School of Medicine, 2-1 Seiryo-machi, Aoba-ku, Sendai, 980-8575 Japan; 3 Department of Diagnostic Radiology, Tohoku University Graduate School of Medicine, 1-1 Seiryo-machi, Aoba-ku, Sendai, 980-8574 Japan; 4 Research Division for Development of Anti-Infective Agents, Institute of Development, Aging and Cancer, Tohoku University, 4-1 Seiryo-machi, Aoba-ku, Sendai, 980-8575 Japan

**Keywords:** Mucormycosis, Pulmonary eosinophilia, *Cunninghamella bertholletiae*

## Abstract

**Background:**

Fungi can cause a variety of infectious diseases, including invasive mycosis and non-invasive mycosis, as well as allergic diseases. The different forms of mycosis usually have been described as mutually exclusive, independent entities, with few descriptions of overlapping cases. Here, we describe the first reported case of a patient with the complication of pulmonary eosinophilia in the course of invasive mucormycosis.

**Case presentation:**

A 74-year-old Japanese man with asthma-COPD overlap underwent emergency surgery for a ruptured abdominal aortic aneurysm. The surgery was successful, but fever and worsening dyspnea appeared and continued from postoperative day (POD) 10. A complete blood count showed leukocytosis with neutrophilia and eosinophilia, and the chest X-ray showed consolidation of the left upper lung at POD 15. We suspected nosocomial pneumonia together with an exacerbation of the asthma-COPD overlap, and both antibiotics and bronchodilator therapy were initiated. However, the symptoms, eosinophilia and imaging findings deteriorated. We then performed a bronchoscopy, and bronchoalveolar lavage (BAL) fluid analysis revealed an increased percentage of eosinophils (82% of whole cells) as well as filamentous fungi. We first suspected that this was a case of allergic bronchopulmonary mycosis (ABPM) caused by *Aspergillus* infection and began corticosteroid therapy with an intravenous administration of voriconazole at POD 27. However, the fungal culture examination of the BAL fluid revealed mucormycetes, which were later identified as *Cunninghamella bertholletiae* by PCR and DNA sequencing. We then switched the antifungal agent to liposomal amphotericin B for the treatment of the pulmonary mucormycosis at POD 29. Despite replacing voriconazole with liposomal amphotericin B, the patient developed septic shock and died at POD 39. The autopsy revealed that filamentous fungi had invaded the lung, heart, thyroid glands, kidneys, and spleen, suggesting that disseminated mucormycosis had occurred.

**Conclusions:**

We describe the first reported case of pulmonary mucormycosis with pulmonary eosinophilia caused by *Cunninghamella bertholletiae*, which resulted in disseminated mucormycosis. Although it is a rather rare case, two important conclusions can be drawn: i) mycosis can simultaneously cause both invasive infection and a host allergic reaction, and ii) *Cunninghamella bertholletiae* rarely infects immunocompetent patients.

## Background

Fungi can cause a variety of infectious diseases, including invasive mycosis and non-invasive mycosis, as well as allergic diseases. The different forms of mycosis are usually described as mutually exclusive, independent entities, with few descriptions of cases that overlap. Mucormycosis is the third most common invasive mycosis. In immunosuppressed patients, this condition can cause non-specific symptoms that include cough, shortness of breath, and chest pain. The fungi can grow very aggressively, destroying the tissue in cases of angioinvasive infection, resulting in dissemination to systemic organs [1, 2]. However, no case presenting the complication of allergic disease in the course of invasive mycosis caused by mucormycetes has been reported. Here, we describe the first reported case of invasive pulmonary mucormycosis presenting with pulmonary eosinophilia.

## Case presentation

**Fig. 1:**
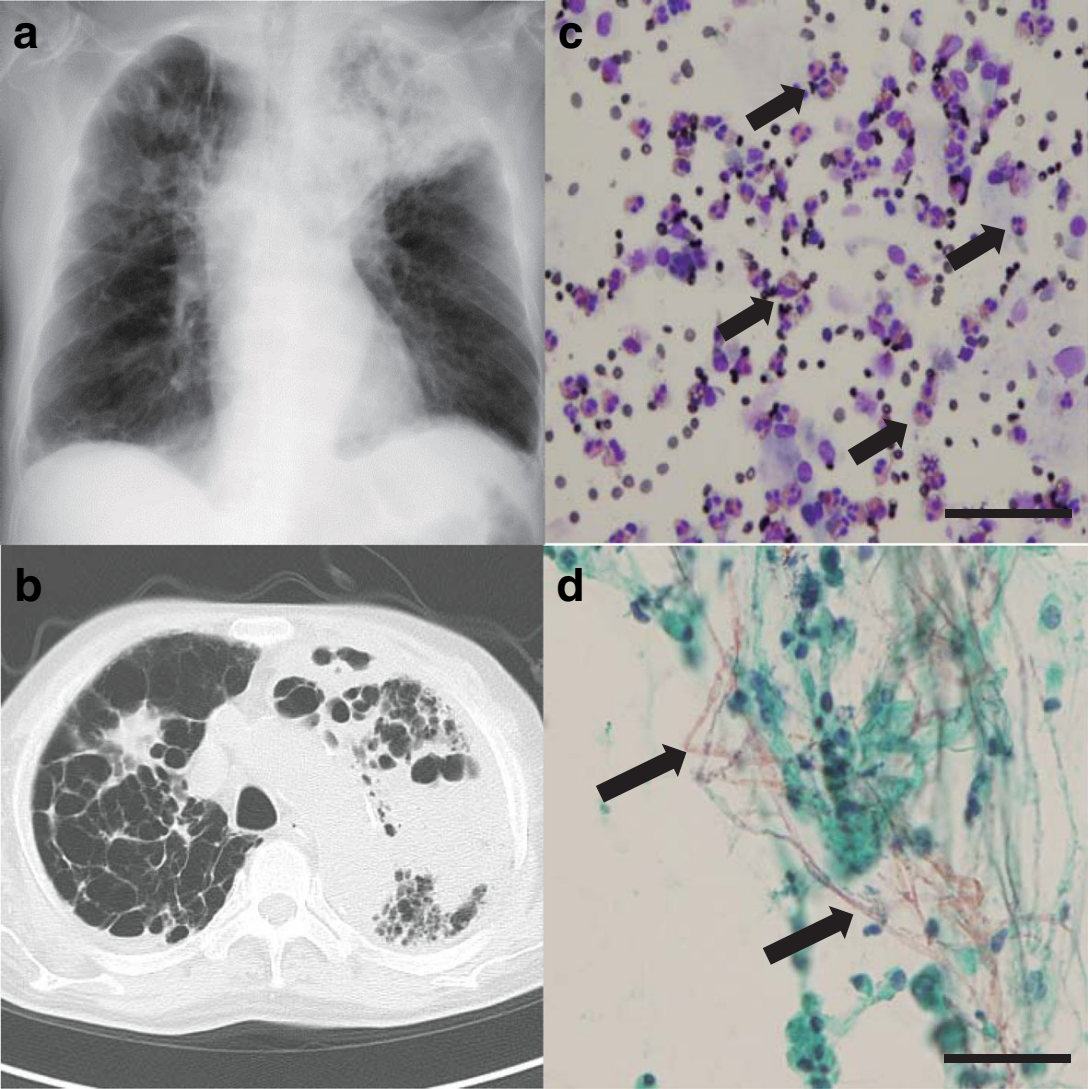
**a**, **b** Chest X-ray (**a**) and computed tomography (**b**) on POD 25 showed massive consolidation in the left upper lobe and a nodular shadow in the right upper lobe. **c** Eosinophilic infiltration (arrow) was confirmed by BAL fluid cytology using Diff-Quik stain. **d** Filamentous fungi were observed in the BAL fluid cytology with Papanicolaou staining

A 74-year-old Japanese man who had well-controlled asthma-COPD overlap (ACO) as a comorbidity and who was being treated with an inhaled corticosteroid plus a long-acting inhaled beta agonist was admitted for emergency surgery to treat a ruptured abdominal aortic aneurysm. The surgery was successful, but fever and worsening dyspnea appeared and continued from postoperative day (POD) 10. Chest auscultation revealed a bilateral wheeze on expiration. A complete blood count at POD 15 showed leukocytosis (12.7 × 10^9^/L) with neutrophilia (10.2 × 10^9^/L) and eosinophilia (1.0 × 10^9^/L). The chest X-ray showed consolidation of the left upper lung. The patient produced mucopurulent sputum in which methicillin-resistant *Staphylococcus aureus* was detected. We suspected nosocomial pneumonia together with an exacerbation of ACO. Thus, we administered meropenem and teicoplanin together with bronchodilator therapy. However, the symptoms did not improve, and the eosinophilia worsened (2.0 × 10^9^/L), although total leukocytes decreased (7.2 × 10^9^/L). The chest X-ray and CT showed worsened consolidation in the left upper lobe at POD 25 (Fig. 1a,b). To examine the cause of refractory pneumonia and to determine the appropriate empiric antibiotic therapy, we performed a bronchoscopy at POD 26. Bronchoalveolar lavage (BAL) fluid analysis showed increased eosinophils (82% in whole cells) and filaments of fungi in the Papanicolaou staining (Fig. 1c,d). We first suspected allergic bronchopulmonary mycosis (ABPM) caused by *Aspergillus* based on published criteria [3] given that the patient exhibited i) bronchial asthma as a comorbidity; ii) an increase in eosinophils in both the serum and BAL fluid; iii) an increase in serum total IgE concentration (2,092 IU/mL); iv) positivity for specific IgE antibodies against fungi, including *Aspergillus* (Table 1), as well as specific IgG antibodies against *Aspergillus* in the serum; and v) filamentous fungi in the BAL fluid. Therefore, we administered corticosteroid therapy (prednisolone; 1 mg/kg) with an intravenous administration of voriconazole at POD 27. However, the fungal culture examination of BAL fluid revealed mucormycetes, which were later identified as *Cunninghamella bertholletiae* by PCR and DNA sequencing. We then switched the antifungal therapy to liposomal amphotericin B for the treatment of the pulmonary mucormycosis at POD 29. Despite replacing voriconazole with liposomal amphotericin B, the patient developed septic shock and died at POD 39.

**Table 1:** Levels of total IgE and fungi-specific IgE antibodies

	Levels	Class^a^
Total IgE	2,092 IU/mL	NA
*Aspergillus*-specific IgE	2.11 UA/mL	Class 2
Mucormycetes-specific IgE	5.18 UA/mL	Class 3
*Candida*-specific IgE	27.4 UA/mL	Class 4
*Alternaria*-specific IgE	0.59 UA/mL	Class 1
*Cladosporium*-specific IgE	0.93 UA/mL	Class 2

The serum levels of total IgE and specific IgE were measured using a fluorescent enzyme immunoassay. ^a^CLASS 0: < 0.35 UA/mL, considered negative for specific IgE; CLASS 1: 0.35–0.7 UA/mL; CLASS 2: 0.71–3.5 UA/mL; CLASS 3: 3.51–17.5 UA/mL; CLASS 4: 17.51–50 UA/mL; CLASS 5: 50.01–100 UA/mL; CLASS 6: > 100 UA/mL. NA: not applicable

**Fig. 2:**
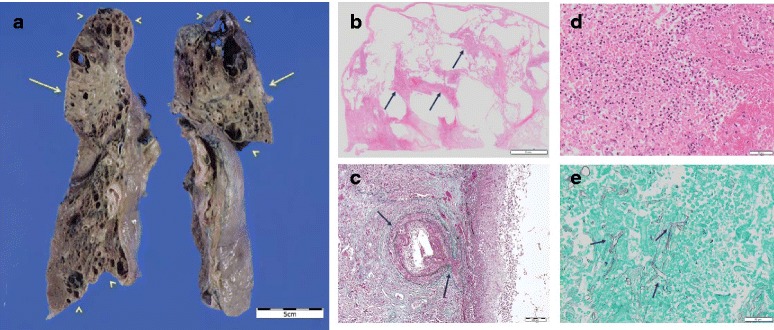
Histopathological findings in the lung tissue. **a** Macroscopic examination revealed severe emphysematous lesions (arrowheads) and massive necrotic lesions (bar = 5 cm). **b** Low-magnification image of a hematoxylin and eosin (HE) stained specimen (bar = 5 mm). In the background of the severely emphysematous lesions, massive infiltration of inflammatory cells and necrosis (arrow) with destruction of the alveoli and vascular walls was observed. **c** Elastica–Masson staining (bar = 200 μm). Destruction of the vascular walls (arrow) was observed. **d** High-magnification image of an HE-stained specimen (bar = 50 μm). Massive infiltration of eosinophils and neutrophils was observed. **e** Grocott staining revealed many filamentous fungi with irregular non-septate hyphae branching at wide angles in the lung lesion (arrow; bar = 50 μm)

**Fig. 3:**
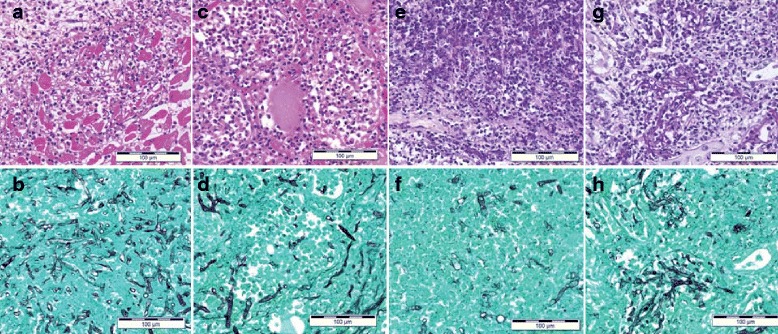
Histological findings in the (**a**, **b**) heart, (**c**, **d**) thyroid gland, (**e**, **f**) spleen, and (**g**, **h**) kidney. HE staining (**a**, **c**, **e**, **g**) and Grocott staining (**b**, **d**, **f**, **h**) are shown (bar = 100 μm). Filamentous fungi with the infiltration of inflammatory cells were also observed in these organs

The autopsy revealed macroscopically extensive necrosis with severe emphysema in both lungs (Fig. 2a). The histopathological examination showed a massive infiltration of inflammatory cells, consisting primarily of neutrophils and eosinophils, with broad necrosis and destruction of the alveoli and vascular walls (Fig. 2b,c,d). Numerous filamentous fungi with irregular non-septate hyphae branching at wide angles were detected within the lesions (Fig. 2e). The fungi had also invaded the heart, thyroid glands, kidneys, and spleen (Fig. 3).

## Discussion

Mucormycosis is an infection caused by mucormycetes and has emerged as a life-threatening invasive mycosis, especially in immunocompromised patients [1, 2]. *Cunninghamella bertholletiae* is a species of the genus *Cunninghamella* of mucormycetes and is the most opportunistic human pathogen of this genus, accounting for approximately 7% of all causative pathogens of mucormycosis. It has been reported that infection with *Cunninghamella* species is a significant risk factor for mortality compared with *Rhizopus* species [1]. Similar to the diagnosis of mucormycosis by other mucormycete species, the diagnosis for *Cunninghamella* infection relies on the identification of organisms in tissues by pathological examination, with confirmation by culture examination. The standard culture-based criteria for the identification of these fungi are the culture conditions, the macroscopic morphology of the colonies, and the microscopic morphology of the organisms [4]. *Cunninghamella bertholletiae* is a fast-growing fungus that can grow at room temperature. However, *Cunninghamella bertholletiae* can grow at temperatures above 40 °C, distinguishing it from *Cunninghamella elegans*, which can be found in laboratory specimens as an environmental contaminant. Microscopically, the morphology of this fungus appears as branched sporangiophores that terminate at a swollen, terminal vesicle with spherical, ovoidal, or ellipsoidal sporangioles. However, it was recently reported that the application of PCR and DNA sequencing using cultured specimens and fresh or fixed tissue specimens is highly sensitive and specific compared with standard culture-based methods [5]. *Cunninghamella bertholletiae* can infect various human tissues. It is angioinvasive and can grow very aggressively, causing destruction of the tissue. Based on a PubMed search of the relationship between mucormycosis and eosinophilia, only one case of paranasal mucormycosis presenting with serum eosinophilia could be identified; however, this case was negative for specific IgE antibodies to mucormycetes [6]. Thus, ours is the first reported case of pulmonary mucormycosis showing pulmonary eosinophilia and resulting in lethal disseminated mucormycosis.

The clinical course of this case raises two important issues concerning the management of mucormycosis. First, mucormycosis can simultaneously cause both invasive infection and host allergic reaction with pulmonary eosinophilia. Mycosis can cause a variety of infectious diseases, including invasive and non-invasive mycosis, as well as allergic diseases, such as pulmonary eosinophilia, bronchial asthma and hypersensitivity pneumonitis. According to our PubMed search, only a few case reports have reported the complication of pulmonary eosinophilia in the course of invasive mycosis [7–10]. The pathogens in these five cases were *Aspergillus* or *Cryptococcus*. One case had chronic granulomatous disease, but the four other patients were immunocompetent. Two cases were reported to be positive for specific IgE antibodies to the pathogenic fungus. There have been no case reports published showing the complication of pulmonary eosinophilia in the course of invasive mucormycosis, suggesting that our patient represents the first reported case. The patient in the present report was positive for specific IgE antibodies to mucormycetes during the described admission (Table 1). Therefore, we assume that the reason this patient presented with pulmonary eosinophilia in the course of invasive mucormycosis is that he had already become allergic to mucormycetes and that the subsequent massive exposure from its proliferation (i.e., pulmonary mucormycosis) induced a severe allergic reaction with eosinophilia. Furthermore, a recent scientific report showed that chitin, which forms microfibrils in the cell walls of most fungi, could induce eosinophilia, supporting our hypothesis [11].

The second issue is that *Cunninghamella bertholletiae* can infect immunocompetent patients. According to previous reports, *Cunninghamella bertholletiae* infection usually develops in immunocompromising conditions, including leukaemia, diabetes mellitus, deferoxamine-based therapy, and organ transplantation. However, our patient did not have such risk factors. Only one reported case has described pulmonary infection with *Cunninghamella bertholletiae* in combination with chronic progressive pleural-pulmonary disease, which occurred in a patient with a history of excessive alcohol consumption [12]. Our patient did not have a history of excessive alcohol consumption, suggesting that ours is also the first report of invasive *Cunninghamella bertholletiae* infection in a postoperative immunocompetent patient with ACO. We assume that the infection route was not associated with surgery because the histopathologic findings of the autopsy showed that *Cunninghamella bertholletiae* had invaded various organs but had not the area around the operated aortic lesion, including the Y graft. Recent epidemiological reports suggested that COPD is the most common risk factor for invasive fungal infection in the ICU setting, and the risk of occurrence might be correlated with the grade of COPD [13, 14]. These reports and our case suggest that the inhalation of a pathogenic fungus can result in colonization and proliferation in an emphysematous lung lesion. We do not have any clear evidence as to when the patient was infected with *Cunninghamella bertholletiae*. However, because the patient was already positive for specific IgE antibodies to mucormycetes (Table 1) and had a significantly severe emphysematous lesion that may have locally compromised the immune system within the lung, it is possible that some mucormycetes had already colonized the lungs before the admission. These fungi would then have proliferated in the lungs and subsequently invaded various organs due to the immunosuppression caused by invasive surgery for the sudden aortic rupture.

We first suspected that the patient had ABPM caused by *Aspergillus*, basing this hypothesis on the International Society for Human and Animal Mycology (ISHAM) working group criteria [3], which are based on those of Greenberger PA and Patterson R [15]. Specifically, the patient had bronchial asthma as a comorbidity, an increase in eosinophils in both serum and BAL fluid, an increase in serum total IgE concentration (2,092 IU/mL) and was positive for both *Aspergillus*-specific IgE (Table 1) and IgG antibodies. The observation of filamentous fungi in the BAL fluid was also supportive of *Aspergillus* infection, although it was not a diagnostic criterion. Moreover, based on previous reports showing that *Aspergillus* is a more common fungal cause of ABPM compared to other fungi, including mucormycetes [16, 17], we first diagnosed the patient with *Aspergillus*-inducted ABPM. However, the fungal culture examination of BAL fluid later revealed that the filamentous fungi were mucormycetes, not *Aspergillus*, causing us to doubt the first diagnosis and to change it to pulmonary infection with mucormycetes. Ultimately, histopathological examinations performed as part of the autopsy showed numerous filamentous fungi with features of mucormycetes within the lungs, confirming the diagnosis of invasive pulmonary mucormycosis rather than ABPM caused by mucormycetes. As we also observed significant infiltration of eosinophils accompanied by mucormycosis lesions in the lungs, we concluded that hypersensitivity to mucormycetes, including *Cunninghamella* spp., may have affected the clinical course of this patient.

Some limitations and caveats should be mentioned with respect to this case. First, other potential causes of the hyper-eosinophilia, such as asthma, drugs (e.g., antibiotics), helminth infection, or another primary infection, should be considered. Asthma can cause eosinophilia, but it has been suggested that asthma-associated eosinophilia is usually mild to moderate (e.g., <1.5 × 10^9^/L) [18]. We therefore considered other eosinophilic pulmonary conditions, including fungal hypersensitivity. Helminths are the commonly identified infectious causes of eosinophilia in some parts of the world, although such infections are very rare in Japan. The present case did not have the risk factors of helminth infection (e.g., ingestion of undercooked meat and vegetables). Moreover, histopathological examination during the autopsy did not provide any evidence of helminth infection, suggesting that helminths were not likely the cause of eosinophilia. It is also possible that antibiotics caused the eosinophilia, which was first observed after initiating antibiotic therapy. However, as the eosinophilia persisted after discontinuation of antibiotics at POD 27, we conclude that this possibility is unlikely. Infectious agents other than mucormycetes can cause leukocytosis and eosinophilia. However, we do not consider this possibility likely given that i) empiric antibiotic therapy did not improve the eosinophilia and ii) a histopathological examination performed as part of the autopsy showed numerous filamentous fungi with features of mucormycetes and significant eosinophil infiltration. These findings suggest that mucormycetes, not other pathogens, were the cause of the pulmonary and systemic eosinophilia. Second, it should also be considered that the corticosteroid therapy may have contributed to the fatal dissemination of mucormycosis. Lastly, there have been reports of patients with early pulmonary mucormycosis infection who were cured with lobectomies [19–21]. However, in many reported cases, the resection of lesions infected with *Cunninghamella bertholletiae* was limited by i) thrombocytopenia associated with underlying malignancy, ii) disseminated infection or multilobular lung involvement at the time of diagnosis, or iii) very late diagnosis in a clinically unstable patient [4]. In our case, surgical intervention was not possible due to both the unstable condition of the patient and multilobular lung involvement at the time of diagnosis.

## Conclusions

In summary, we describe here the first reported case of pulmonary mucormycosis with pulmonary eosinophilia caused by *Cunninghamella bertholletiae*, which resulted in disseminated mucormycosis. Although it is a rather rare case, it has provided us with the important clinical insight that mycosis, including invasive mucormycosis, can present with significant eosinophilia and pulmonary eosinophilic infiltration.

## Abbreviations

ABPM: Allergic bronchopulmonary mycosis
ACO: Asthma-COPD overlap
BAL: Bronchoalveolar lavage
COPD: Chronic obstructive pulmonary disease
POD: Postoperative day
